# Multi-Step Pathogenesis and Induction of Local Immune Response by Systemic Candida Albicans Infection in an Intravenous Challenge Mouse Model

**DOI:** 10.3390/ijms150814848

**Published:** 2014-08-22

**Authors:** Voon-Kin Chin, Kuan-Jeang Foong, Abdullah Maha, Basir Rusliza, Mohtarrudin Norhafizah, Pei Pei Chong

**Affiliations:** 1 Department of Biomedical Science, Faculty of Medicine and Health Sciences, Universiti Putra Malaysia, Selangor 43400, Malaysia; E-Mail: darkangel_wuu@hotmail.com; 2 Department of Pathology, Faculty of Medicine and Health Sciences, Universiti Putra Malaysia, Selangor 43400, Malaysia; E-Mails: maha@upm.edu.my (A.M.); norhafizahm@upm.edu.my (M.N.); 3 Department of Human Anatomy, Faculty of Medicine and Health Sciences, Universiti Putra Malaysia, Selangor 43400, Malaysia; E-Mail: rusliza@upm.edu.my; 4 Translational Infectious Diseases Program, Centre for Translational Medicine, Department of Microbiology, National University of Singapore, Level 15 MD6, 5 Science Drive 2, National University of Singapore, Singapore 117597, Singapore

**Keywords:** *Candida albicans*, erythropoietin (EPO), pattern recognition receptors (PRRs), cytokines, chemokines, red blood cells and haemoglobin

## Abstract

Different murine species differ in their susceptibility to systemic infection with *Candida albicans*, giving rise to varied host immune responses, and this is compounded by variations in virulence of the different yeast strains used. Hence, this study was aimed at elucidating the pathogenesis of a clinical *C. albicans* isolate (HVS6360) in a murine intravenous challenge model by examining the different parameters which included the counts of red blood cells and associated components as well as the organ-specific expression profiles of cytokines and chemokines. Kidneys and brains of infected mice have higher fungal recovery rates as compared to other organs and there were extensive yeast infiltration with moderate to severe inflammation seen in kidney and brain tissues. Red blood cells (RBCs) and haemoglobin (Hb) counts were reduced throughout the infection period. Pattern recognition receptors (PRRs), chemokines and cytokine transcription profiles were varied among the different organs (kidney, spleen and brain) over 72 h post infections. Transcription of most of the PRRs, cytokines and chemokines were suppressed at 72 h post infection in spleen while continuous expression of PRRs, cytokines and chemokines genes were seen in brain and kidney. Reduction in red blood cells and haemoglobin counts might be associated with the action of extracellular haemolysin enzyme and haeme oxygenase of *C. albicans* in conjunction with iron scavenging for the fungal growth. Renal cells responsible for erythropoietin production may be injured by the infection and hence the combined effect of haemolysis plus lack of erythropoietin-induced RBC replenishment leads to aggravated reduction in RBC numbers. The varied local host immune profiles among target organs during systemic *C. albicans* infection could be of importance for future work in designing targeted immunotherapy through immunomodulatory approaches.

## 1. Introduction

*Candida albicans* is a major fungal pathogen that causes both mucocutaneous and disseminated infections, particularly in debilitated or immunocompromised patients [1]. *C. albicans* exhibits immense plasticity in its ability to colonize various microniches within the human host, thus causing a wide range of infections. Systemic infection with *Candida* species, particularly *C. albicans* often involves dissemination to multiple internal organs via hematogenous spread and is often life threatening, with the overall prognosis comparable to a septic shock and multiple organ failure [2]. Invasive *Candida* infections are not only increasing in incidence in intensive care units of hospitals, they also present problems in terms of treatment and management [3]. Systemic *Candida* infection is typified by three stages, namely dissemination via the bloodstream, exit from the bloodstream via traversal of the endothelium and infection of deep-seated organs. The epidemiology of the infections could be attributed to contaminated vascular catheters or parenteral nutrition tubings and also other biomedical devices which have *Candida* biofilm growth. 

*In vitro*, *in vivo* as well as *ex vivo* infection models have been adopted by different researchers for studying various aspects of systemic candidiasis. For *in vivo* models, murine intravenous challenge models are frequently used for investigating mechanisms of candidal virulence, host defense and evaluation of new antifungal agents [4] and is a well characterized model of severe clinical disseminated infection. In this model, major target organs for colonization are the kidney, brain and heart [5]. The primary end-point of systemic candidiasis in a mouse model is mortality but the underlying reason has not been clearly ascertained, although one likely reason is kidney failure due to acute fungal pyelonephritis [4]. The murine intravenous challenge model is well suited to studies on transcriptional profiling of systemic and invasive candidiasis due to its similarities to severe human disseminated infection.

There has been a spike in the number of papers in the past 7–8 years reporting the global transcriptional response of selected organs or cell types in candidiasis models profiled using microarray technology. Although microarray profiles can cast a wide net for the highest possible number of different gene transcripts examined, sometimes the expression changes of low-expression transcripts may be masked by the high-abundance transcripts, and the expression-fold data may not be reliable. 

There are vast inter-species as well as intra-species and inter-strain variations of *Candida* in terms of virulent determinants and infection potential. Theoretically, it is possible for a strain which causes mucosal infections to be also able to cross the endothelial barrier, colonise and infect the deep-seated organs, leading to disseminated infections. Indeed, it has been noted that these *Candida* strains could be transmitted through the hands of healthcare workers who are handling central venous catheters [6]. Thus, we are interested in probing the virulence of a strain that causes mucosal infection in an *in vivo* systemic candidiasis setting and to observe the immune response mounted by the host against the originally mucosal derived strain.

Hence, this study aims to investigate pathogenesis of systemic candidiasis using an isolate that originated from a mucosal site to establish a murine systemic candidiasis model. We studied the effect on the red blood cells and erythropoiesis as the fungus invades the bloodstream and elucidated the gene expression changes of the whole kidney, brain and spleen in response to *C. albicans* infection using a quantitative reverse transcriptase polymerase chain reaction (qRT-PCR) array platform. 

## 2. Results and Discussion

### 2.1. Quantitative Yeast Count

**Figure 1:**
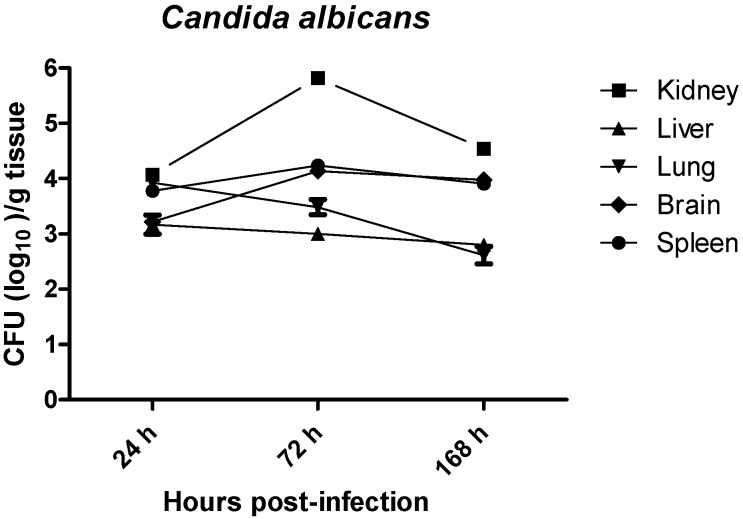
Growth of *C. albicans* in tissues of immuncompetent BALB/C female mice. Mice were inoculated with *C. albicans* (5 × 10^5^ organisms/mouse). At specific time intervals post infection, mice were euthanized and the recovery of *C. albicans* from lungs, livers, kidneys, spleens and brains were quantified by culturing the tissue homogenates on Sabouraud dextrose agar (SDA agar). Results represent the mean ± SD of log_10_ CFU/gram of tissues of three mice per time intervals. CFU: colony forming unit.

The tissue distribution of *C. albicans* in systemically infected BALB/c female mice was determined by culturing the kidneys, lungs, livers, spleens and brains homogenates at 24, 72, and 128 h (1, 3, and 7 days respectively) post infection. As shown in Figure 1, *C. albicans* was successfully recovered from all organs with different growth rates. Fungal loads in lungs and livers were rapidly decreased by nearly 2 logs and 1 log respectively from 24 to 168 h post-infection. Fungal loads in kidneys increased by nearly 2 logs from 24 to 72 h post-infection and decreased slightly at 168 h post-infection. Fungal loads in kidney remains the highest among all organs within the 7 days post-infection. There were no significant changes in the fungal loads in spleen and brain within 7 days post infection and the fungal loads were around 3.5–4.0 log. Taken together, the results showed that the fatal outcome caused by systemic *C. albicans* infection was associated with the logarithmic growth of *C. albicans* in the kidneys and also persistent recovery of *C. albicans* from spleen and brain.

### 2.2. In Vitro Haemolysin Production by C. albicans

*Candida albicans* (HVS6360) showed beta haemolysis with a haemolysis index 2.278. There was presence of translucent, well-defined, ring shaped formation around the colony which indicated the presence of haemolysin activity.

### 2.3. Red Blood Cells and Haemoglobin Counts

**Figure 2:**
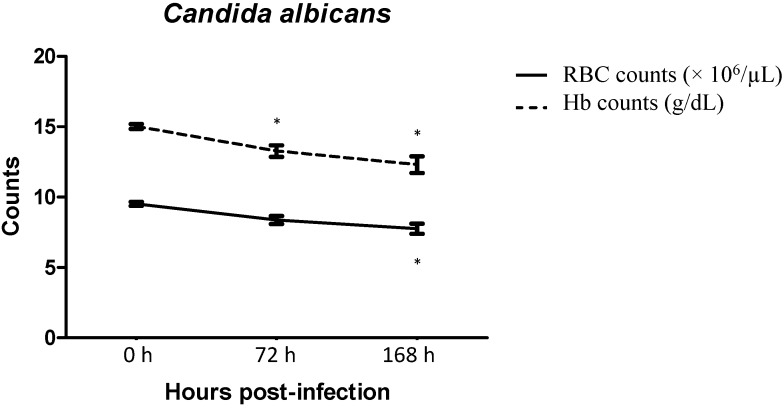
Red blood cell (RBC) and haemoglobin (Hb) counts of mice systemically infected with *C. albicans* (5 × 10^5^ organisms/mouse). At specific time intervals post infection, blood was drawn from mice and subjected to blood analysis. Results represent the mean ± SD of six mice per time intervals. Symbol * indicates the reduction was statistically significant at *p <* 0.05.

As shown in Figure 2, the red blood cell (RBC) and haemoglobin (Hb) counts of mice systemically infected with *C. albicans* were consistently reduced within the 7 days post-infection. The RBC count was reduced approximately 9% at 72 h post-infection followed by a further reduction of 15% at 168 h post infection as compared to 0 h pre-infection. The Hb count was reduced 9.5% at 72 h post infection and approximately 15% at 168 h post infection as compared to 0 h pre-infection. The reduction in RBC counts at 168 h and reduction in haemoglobin counts at 72 and 168 h were statistically significant (*p <* 0.05). 

### 2.4. Murine Erythropoietin Expression

**Figure 3:**
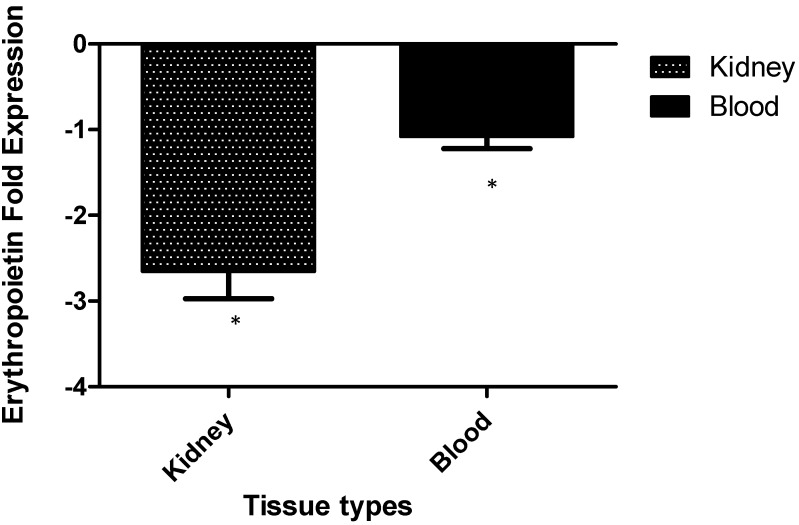
Temporal expression of erythropoietin mRNAs from blood and kidney of *C.albicans*-infected mice at 72 h post-infection. Transcript levels for erythropoietin were quantified by real-time reverse transcription polymerase chain reaction (RT-PCR). Bar graph represents average expression of each gene normalized to the reference genes, β-actin and Glyceraldehyde 3-phosphate dehydrogenase (GAPDH), to attain a normalized gene expression ratio. Results were means of three biological replicates and two technical replicates for each biological replicate. Bars indicate standard error of mean and * revealed significant differences at the level of *p* < 0.05 as compared with control group, respectively. The fold changes were calculated based on the normalization of the target gene with the average of housekeeping genes (Beta actin and GAPDH) using the ΔΔ*C*_t_ method in infected mice as compared to that in uninfected mice.

As shown in Figure 3, the transcript level of erythropoietin (EPO) in mice systemically infected with *C. albicans* at 72 h post infection was significantly down regulated for more than two folds from kidney (*p* = 0.0008) and one fold from blood (*p* = 0.0028) as compared to uninfected control.

### 2.5. Histopathology

**Figure 4:**
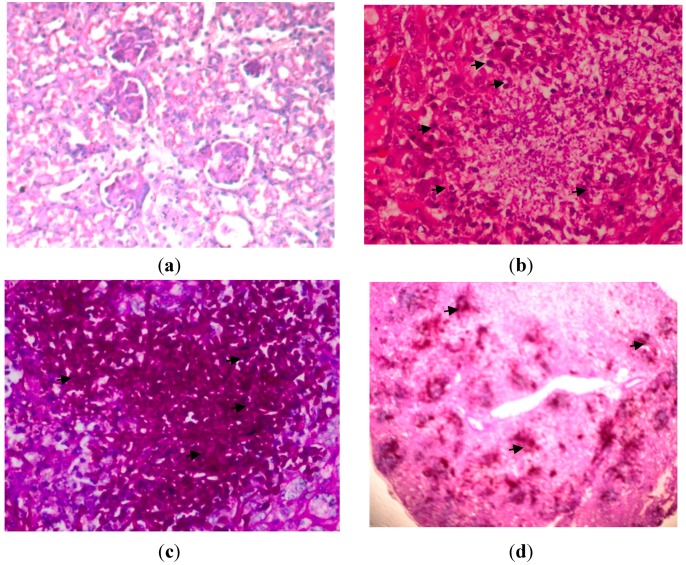
Kidney tissues on 72 h post-infection with *C. albicans*, (**a**) uninfected kidney; Haematoxylin-eosin (H&E) stain; original magnification, ×400 (Tissues were normal, no congestion of tubules, inflammation, haemorrhage and glomeruli); (**b**) H&E stain; original magnification, ×400 (Arrows indicated a large polymorphonuclear infiltrate is demonstrated in the abscess in the center of the photomicrograph); (**c**) Periodic acid Schiff (PAS) stain; original magnification, ×400 (Arrows indicated hyphae and fungal bodies of *C. albicans*); and (**d**) PAS stain; original magnification, ×40 (Arrows indicate that here there is confluent invasion of the hyphae and fungal bodies in the kidney).

The microscopic results showed there were masses of branched, septate hyphae, in addition to pseudohyphae and round to oval budding yeast cells (blastospores) in kidney and brain. There was extensive infiltration of *C. albicans* in kidney tissues. The fungal components were found much higher in the cortex, particularly prominent in the proximal and distal tubules than in the medulla. There were signs of congestion, hemorrhages, tubular degeneration and neutrophil infiltration in the kidneys. Necrosis and abscess formation were evident with moderate to severe inflammation (Figure 4).

**Figure 5:**
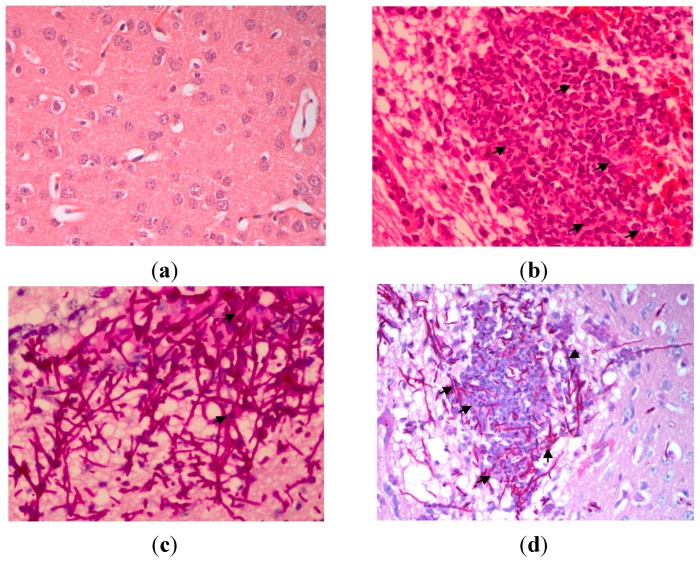
Brain tissues on 72 h post-infection with *C. albicans*, (**a**) uninfected brain; H&E stain; original magnification, ×400 (Tissues were normal, no inflammation, haemorrhage and infiltration of polymorphonuclear cells were observed) (**b**) H&E stain; original magnification, ×400 (Arrows indicate a large polymorphonuclear infiltrate is demonstrated in the abscess in the center of the photomicrograph); (**c**) PAS stain; original magnification, ×400 (Arrows indicated hyphae and fungal bodies of *C. albicans*); and (**d**) PAS stain; original magnification, ×400 (Arrows indicate hyphae and fungal bodies of *C. albicans* surrounding the abscess).

Meanwhile, yeast infiltration and tissue pathology of mice infected with *C. albicans* in brain was moderate. Hyphae and yeast cells can be seen throughout the brain parenchyma. Both true hyphae and yeast cells were distributed in the white and gray matters of the brain and cerebellum. Mild to moderate inflammation was observed in the brain parenchyma and around the blood vessels. Petechial haemorrhage was observed in the brain parenchyma and cerebrum cortex. There was a mild meningeal congestion of the brain. In addition, micro-abscesses comprised of neutrophils were also detected in the parenchyma (Figure 5).

**Figure 6:**
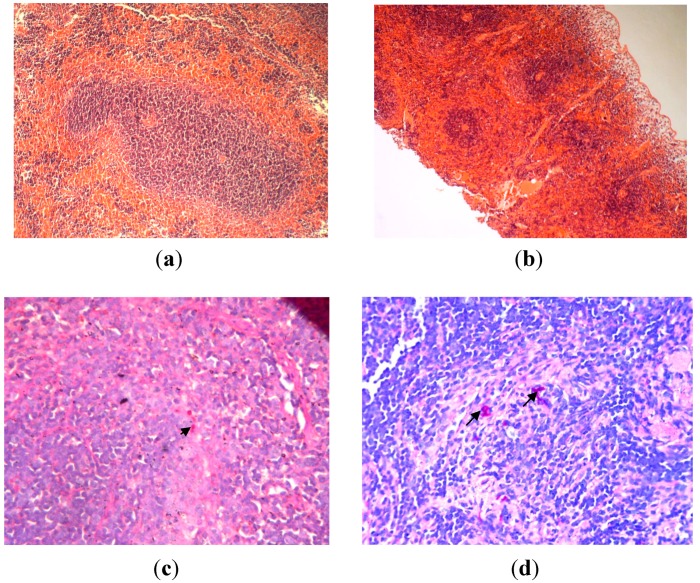
Spleen tissues on 72 h post-infection with *C. albicans*, (**a**) uninfected spleen; H&E stain; original magnification, ×400 (There is no architecture loss, necrosis or polymorphonuclear infiltration in the spleen tissue) (**b**) H&E stain; original magnification, ×40 (Spleen tissue appear normal with no evidence of tissue pathology); (**c**) PAS stain; original magnification, ×400 (Arrows indicated few hyphae and fungal bodies of *C. albicans* observed); (**d**) PAS stain; original magnification, ×400 (Arrows indicate few hyphae and fungal bodies of *C. albicans* observed).

Although there were yeast cells detected in spleen, however, there was no evidence of tissue pathology observed as there was no architectural loss of the organs, and no inflammation, hemorrhage, abscess/microabscess formation or necrosis was observed (Figure 6). 

### 2.6. Pattern Recognition Receptor (PRR) Gene Expression during Infection

The PRR genes expression for kidney, brain and spleen is shown in Table 1. In kidney, most of the PRR genes examined in this study showed upregulation in response to *C. albicans* infection. TLR2 (Toll-like receptor 2), Clec7a/Dectin-1, Clec4n/Dectin-2 and Clec4e/Mincle expressions were statistically up-regulated at 24 h as well as 72 h post infection while, TLR4 was statistically up-regulated only at 72 h post infection. TLR9 expression was increased at 24 h post infection. For the mannose receptors, Mrc1 expression was up-regulated but Mrc2 expression was suppressed at 24 and 72 h post infection. The suppression of Mrc2 was statistically significant at 24 h post infection.

**Table 1:** The fold changes of pattern recognition receptors (PRRs) in whole kidney, spleen and brain towards systemic *C. albicans* infection at 24 and 72 h post-infection. The fold changes were calculated based on the normalization of the target gene to the reference genes, β-actin, GAPDH, 18 s ribosomal RNA and beta-2 microglobulin using the ΔΔ*C*_t_ method in infected mice as compared to that in uninfected mice. Results were means of fold changes of three biological replicates and highlighted parts are the genes which were statistically significant at *p* < 0.05 as compared with uninfected group, respectively.

Organ	Kidney		Brain		Spleen	
Gene	Time					
	24 h Mean Fold Changes/*p* Values	72 h Mean Fold Changes/*p* Values	24 h Mean Fold Changes/*p* Values	72 h Mean Fold Changes/*p* Values	24 h Mean Fold Changes/*p* Values	72 h Mean Fold Changes/*p* Values
Tlr2	13.03/0.0022	10.58/0.0002	3.71/0.0092	7.18/0.00007	1.96/0.023	1.19/0.194
Tlr4	1.51/0.108	3.94/0.006	1.27/0.087	3.11/0.0012	−1.01/0.88	1.09/0.589
Tlr9	2.11/0.145	1.01/0.807	−1.61/0.123	−4.78/0.012	−1.59/0.002	−3.77/0.008
Mrc1	−2.39/0.208	2.00/0.235	−1.50/0.097	−1.92/0.04	−1.22/0.146	3.15/0.00002
Mrc2	−2.53/0.0073	1.62/0.053	2.09/0.264	−1.33/0.291	−1.44/0.188	1.03/0.924
Clec7a	6.48/0.0061	4.18/0.04	1.66/0.410	1.95/0.148	−1.17/0.251	−2.45/0.035
Clec4n	18.89/0.0018	40.63/0.036	4.48/0.0004	17.12/0.00009	2.40/0.0004	−1.04/0.933
Clec4e	24.41/0.00181	75.51/0.0017	8.02/0.08	20.93/0.00011	6.75/0.003	6.63/0.00074

In the brain, only TLR2 and Clec4n/Dectin-2 were statistically up-regulated at 24 h post infection while TLR2, TLR4, Clec4n/Dectin-2 and Clec4e/Mincle were statistically up-regulated at 72 h post infection. Both TLR9 and Mrc1 expression levels were suppressed with suppression at 72 h post infection being statistically significant. Mrc2 expression was increased at 24 h but was suppressed at 72 h post infection. 

In spleen, most of the PRR genes were suppressed at 72 h post infection with just Mrc1 and Clec4e/Mincle being statistically significantly up-regulated. Meanwhile, TLR2, Clec4n/Dectin-2 and Clec4e/Mincle expressions were increased statistically significantly at 24 h post infection. Fold changes of the PRR genes were highest in kidney, followed by brain and spleen.

### 2.7. Cytokine Gene Expression during Infection

The cytokine genes expression for kidney, brain and spleen are shown in Table 2. Most of the cytokines examined in the kidney showed increased expression in response to *C. albicans*. The proinflammatory cytokine genes, Interleukin-6 (IL-6) and Tumour Necrosis Factor-alpha (TNF-alpha), which had been linked to the acute-phase response, as well as the T cell related genes, such as IL-23a, IL-12a and inhibitory cytokines such as IL-10 and Transforming Growth Factor-beta were statistically up-regulated at 24 h post infection. TGF-beta, IL-23a, IL-12a, IL-10 and TNF alpha were statistically up-regulated at 72 h post infection. Meanwhile, Th1 response cytokine, IFN-gamma expression and Th17 response cytokine, IL-17a were not statistically increased at 24 and 72 h post infection whereas Th2 response cytokine, IL-4 was not statistically suppressed at 24 and 72 h post infection. IL-13 expression did not show any statistically significant increase at both 24 and 72 h post infection. 

**Table 2:** The fold changes of cytokine genes in whole kidney, spleen and brain towards systemic *C. albicans* infection at 24 and 72 h post-infection. The fold changes were calculated based on the normalization of the target gene to the reference genes, β-actin, GAPDH, 18s ribosomal RNA and beta-2 microglobulin using the ΔΔ*C*_t_ method in infected mice as compared to that in uninfected mice. Results were means of fold changes of three biological replicates and highlighted parts are the genes which were statistically significant at *p* < 0.05 as compared with uninfected group, respectively.

Organ	Kidney		Brain		Spleen	
Gene	Time					
	24 h Mean Fold Changes/*p* Values	72 h Mean Fold Changes/*p* Values	24 h Mean Fold Changes/*p* Values	72 h Mean Fold Changes/*p* Values	24 h Mean Fold Changes/*p* Values	72 h Mean Fold Changes/*p* Values
Tgfbi	3.62/0.0012	21.71/0.001	2.21/0.016	7.90/0.00011	1.81/0.0006	2.08/0.051
Il10	6.23/0.0019	121.66/0.048	6.82/0.18	46.35/0.00002	6.44/0.0018	21.16/0.031
Tnf	65.04/0.0006	36.93/0.002	12.61/0.055	58.13/0.0005	−1.57/0.173	−3.02/0.012
Il13	1.35/0.8851	1.49/0.734	3.90/0.227	1.12/0.863	2.22/0.355	1.24/0.853
Ifng	12.32/0.1147	−2.14/0.059	6.71/0.202	1.99/0.447	−1.02/0.829	−8.40/0.00002
Il23a	8.07/0.0013	21.51/0.0006	3.32/0.245	3.19/0.008	4.26/0.021	1.21/0.818
Il6	35.17/0.016	49.15/0.056	15.78/0.005	83.93/0.0001	1.36/0.466	−1.65/0.918
Il4	−1.54/0.545	−1.00/0.923	5.66/0.121	−1.04/0.776	−1.15/0.689	−2.54/0.0011
Il17a	1.08/0.980	1.43/0.446	10.80/0.168	3.10/0.029	7.10/0.008	−1.19/0.527
Il12a	20.87/0.0037	80.26/0.017	1.77/0.174	5.06/0.0008	−1.35/0.120	1.08/0.671

In brain, only IL-6 and TGF-beta showed statistically significant up-regulation in the brain at 24 h post infection while IL-6, TNF-alpha, IL-10, IL-23a, IL-12a, IL-17a and TGF-beta were significantly up-regulated at 72 h post infection in the brain. IFN-gamma expression was not increased at 24 and 72 h post infection. IL-4 expression did not exhibit any significant changes at 24 and 72 h post infection while IL-13 increased slightly albeit not to a statistically significant level at 24 and 72 h post infection.

Spleen showed minimal changes in the cytokines expression level in response to *C. albicans* infection. Cytokines such as IL-17a, TGF-beta, IL-23a and IL-10 were significantly up-regulated at 24 h post infection. The expression of most of the cytokines was suppressed, with the downregulation of IFN-gamma, TNF-alpha, and IL-4 being statistically significant at 72 h post infection and with the exception of IL-10 which was up-regulated at a statistically significant level. 

### 2.8. Chemokine Gene Expression during Infection

The chemokine genes expression for kidney, brain and spleen are shown in Table 3. In kidney, all the chemokines showed increased expression with Cxcl-1, Cxcl-10, Cxcl-11 and Ccl5 being significantly increased at 24 h post infection. On the other hand, at 72 h post infection, only Cxcl-1, Csf2, and Cxcl-11 expression were increased with Cxcl-1 being significantly up-regulated. The expression of other chemokines was suppressed, but not to a statistically significant extent. 

**Table 3:** The fold changes of chemokine genes in whole kidney, spleen and brain towards systemic *C. albicans* infection at 24 and 72 h post-infection. The fold changes were calculated based on the normalization of the target gene to the reference genes, β-actin, GAPDH, 18s ribosomal RNA and beta-2 microglobulin using the ΔΔC*_T_* method in infected mice as compared to that in uninfected mice. Results were means of fold changes of three biological replicates and highlighted parts are the genes which were statistically significant at *p* < 0.05 as compared with uninfected group, respectively.

Organ	Kidney		Brain		Spleen	
Gene	Time					
	24 h Mean Fold Changes/*p* Values	72 h Mean Fold Changes/*p* Values	24 h Mean Fold Changes/*p* Values	72 h Mean Fold Changes/*p* Values	24 h Mean Fold Changes/*p* Values	72 h Mean Fold Changes/*p* Values
Cxcl1	104.21/0.0235	126.02/0.0007	64.59/0.077	27.81/0.0002	6.89/0.001	−1.43/0.950
Cxcl10	76.82/0.024	−1.03/0.951	6.63/0.015	1.28/0.361	2.57/0.00006	−17.84/0.003
Ccl5	17.43/0.016	1.30/0.651	−13.36/0.047	−8.22/0.049	−1.77/0.00006	−5.45/0.0005
Cxcl9	29.72/0.140	−2.14/0.322	1.49/0.442	−3.69/0.051	3.08/0.0005	−13.24/0.004
Csf2	1.21/0.583	3.53/0.356	−1.02/0.473	−1.88/0.338	−2.04/0.730	1.18/0.487
Cxcl11	56.75/0.019	1.95/0.100	6.25/0.036	2.44/0.10	1.50/0.304	−2.93/0.123
Ccl12	11.71/0.074	−1.20/0.644	32.37/0.022	−13.63/0.04	23.22/0.006	2.02/0.235

In brain, Cxcl-1, Cxcl-9, Cxcl-10, Cxcl-11 and Ccl12 expression were increased with Cxcl-10, Cxcl-11 and Ccl-12 expression being significantly up-regulated while Csf2 and Ccl5 expression were suppressed with Ccl5 suppression being statistically significant at 24 h post infection. Cxcl-1, Cxcl-10 and Cxcl-11 expression were increased with Cxcl-1 expression being significantly up-regulated while Ccl5, Cxcl-9, Csf2 and Ccl12 expression was suppressed with Ccl5 and Ccl12 suppression being statistically significant at 72 h post infection. 

In the spleen, Cxcl-1, Cxcl-10, Cxcl-9, Cxcl-11 and Ccl12 expressions were increased with Cxcl-1, Cxcl-10, Cxcl-9 and Ccl12 expression being statistically significant while Ccl5 and Csf2 expression were suppressed with suppression of Ccl5 being statistically significant at 24 h post infection. At 72 h post infection, Csf2 and Ccl12 expression were increased though not to a significant level whereas Cxcl-1, Cxcl-10, Cxcl-9, Ccl5 and Cxcl11 expression were down regulated with that of Cxcl-10, Ccl5, and Cxcl-9 being statistically significant. Overall, the results indicated that most of the chemokines examined in this study showed increased expression at 24 h post infection followed by down regulated expression at 72 h post infection and this pattern was consistently seen in kidney, spleen and brain tissues. 

### 2.9. Discussion

The present study utilized a clinical isolate of *C. albicans* isolated from the vaginal site of a healthy individual to investigate on how the *C. albicans* as a commensal, can cause life threatening systemic infection in debilitated host in the intravenous challenge murine model. 

Analysis of tissue colonization by quantitative yeast count revealed significant yeast levels in the brain, kidney, lungs, liver and spleen where kidney, brain and spleen were most colonized by *C. albicans*. The differential targeting of *C. albicans* to organs suggests that factors such as differential adherence or resident host defense mechanism regulate the level of colonization in different tissues. This pattern of tissue colonization follows the trend seen in most animal models of intravenous induced systemic candidiasis [7,8].

The presences of hyphae/pseudohyphae in kidney and brain tissues of *C. albicans*-infected mice were associated with a substantial inflammatory response, characterized by neutrophilic cells infiltration. In sharp contrast, fewer yeast cells were observed in spleen tissues of *C. albicans*-infected mice with no inflammatory responses or where damages of tissues were observed. This showed that the formation of hyphae/pseudohyphae that might promote neutrophil infiltration and morphogenesis is an important virulence factor in establishing infection in the mammalian host [9,10].

In this study, *in vitro* production of haemolysin by *C. albicans* was demonstrated. In addition, mice infected systemically with *C. albicans* results in the steady reduction of both the RBC count and haemoglobin level within 7 days of post-infection. Hence, with the findings from both *in vitro* and *in vivo*, we speculate that the reduction of both RBC count and haemoglobin level *in vivo* is likely caused by the action of extracellular haemolysin enzyme of *C. albicans* with the dual function of lysing the RBC and acquiring iron as nutrient in order to establish disseminated infection in the host.

Our studies showed that mice infected systemically with *C. albicans* results in decrement of erythropoietin (EPO) formation in kidney and blood. The decreased production of EPO is possibly caused by damage of the kidney cells which is the primary organ responsible for the erythropoietin production [11]. Some studies have shown that renal peritubular cells are responsible for the erythropoietin production in kidney [12,13]. In malaria infection, involvement of cytokines and other mediators of inflammation lead to decrease in erythropoietin production [14]. However, decreased production of EPO during systemic *C. albicans* infection and its impact on host still remains elusive and should be investigated further.

Previous studies mostly centered on investigating the responses of single host cell types, such as neutrophils [15,16], endothelial cells [17,18] and macrophages [19,20], which cannot reflect the real situation occurring in the affected organs upon *C. albicans* infection. By comparison, our study was designed to assess the kinetics of expression of genes encoding multiple host immune molecules over a 72 h time course to gain more accurate understanding of the dynamic local host responses elicited at various target organs (kidney, spleen and brain) during systemic *C. albicans* infection.

In the present study, there was appreciable change in the genes encoding PRR over 72 h time course of infection in brain and kidney, particularly TLR2, TLR4, Dectin-1, Dectin-2 and Mincle, whilst spleen showed minimal in PRRs expression, particularly TLR2, Dectin-2 and Mincle. The continuous and appreciable expression of PRRs in brain and kidney might be associated with the progression of infection in these sites. However, the cells responsible for the expression of PRRs were not identified in this study. Some studies have shown that renal tubular epithelial cells were responsible for the expression of TLR2 and TLR4 in response to renal [21,22] and microglial cells in brain were responsible for the expression of TLRs and Dectin-1 in microglial-mediated neuro-inflammation and central nervous system (CNS) injury following infection [23,24]. Meanwhile, the minimal in PRRs expression in spleen was most likely due to the lower number of yeast cells that were able to establish an infection in spleen. 

The expression of genes encoding several chemokines associated with the recruitment and activation of phagocytes was evaluated in this study. A previous report [25] on DNA array in human leukocytes expression stimulated with *C. albicans* showed up-regulation of *Mip-1α*, *Mip-1β*, *Mip-2α* and *Mcp-1*. Our data showed most of the chemokines examined in this study were increased locally at an early time-point of 24 h post infection. Spleen, kidney and brain showed different profiles of chemokines in response to systemic *Candida* infection as shown in Table 3. However, there is some commonality such as the expression of Cxcl-1 and Cxcl-10 which were elevated in all three organs at 24 h post infection. On the other hand, some chemokines were only significantly expressed in certain tissues in response to *C. albicans* infection. For example, Ccl5 was only significantly expressed in kidney while Ccl12 was significantly expressed in brain and spleen, Cxcl-11 was significantly expressed in brain and kidney and Cxcl-9 was only significantly expressed in spleen. Cxcl-1 is involved in mobilization of leukocyte infiltrates, especially neutrophil towards the site of infection and Maccalum [8] hypothesized that Cxcl-1 production is a critical early event that mobilizes the host infiltrates seen in the kidney. On the other hand, Ccl12 is known for chemotactic recruitment of monocytes/macrophages to the site of inflammation [26] which suggests that cells of monocytic lineage might contribute to the elevation of Ccl12 in the brain and spleen tissues. Meanwhile, Cxcl-9, Cxcl-10 and Cxcl-11 are interferon-inducible members of the CXC chemokine family which facilitate selective recruitment of mononuclear leukocytes, natural killer cells, and plasmacytoid dendritic cells to sites of inflammation [27]. Ccl5 or RANTES is chemotactic for T cells, eosinophils and basophils. It plays an active role in recruiting leukocytes into inflammatory sites. Ccl5 is involved in a variety of diseases related with the kidney [28,29] and confers protection against parasitic infection [30]. Overall, the findings suggested that induction of chemokines was a direct effect of *C. albicans* exposure which may be beneficial for the early recruitment of inflammatory cells against *C. albicans*. However, the functional roles of these molecules in tissue specific host defense against systemic candidasis should be explored further.

Apart from their role in cellular recruitment, some *in vitro* studies showed that a number of chemokines have been found to mediate direct antimicrobial effects against bacteria, parasites and fungi [31,32]. Hence, chemokines which show enhanced expression towards systemic *C. albicans* infection in this study can be chosen for investigation on their direct antifungal activity towards *C. albicans* both *in vitro* and *in vivo*.

The present study demonstrated that different organs showed different cytokine phenotypes during systemic *C. albicans* infection. At 24 h post infection, Th1/innate/suppressive responses which mainly involved IL-6, TNF-alpha, IL-23a, IL-12a, IL-10 and TGF-beta were observed in kidney, whereas the innate/suppressive responses which involved IL-6 and Tgf-beta were observed in brain and Th17/suppressive responses which mainly involved IL-17a, IL-23a, IL-10 and TGF-beta were observed in spleen. Meanwhile, at 72 h post infection, Th1/innate/suppressive responses which mainly involved IL-12a, TNF-alpha, IL-23a, IL-10 and TGF-beta were observed in kidney, Th1/Th17/innate/suppressive responses which involved IL-6, TNF-alpha, IL-23a, IL-12a, IL-10, IL-17a and TGF-beta were observed in brain and suppressive response by IL-10 and suppression of innate/Th1/Th2 responses which involved TNF-alpha, IFN-gamma and IL-4 was observed in spleen. 

IL-12 is an important cytokine which has links to both innate and adaptive immune system. IL-12 enhances phagocytosis of *C. albicans* blastoconidia and increases oxygen dependent and independent candicidal activity. Moreover, IL-12 is also an essential component of the adaptive response that leads to the generation of Th1-type cytokine responses and protection against disseminated disease [33,34]. On the other hand, IL-23 is another important cytokine which is essential for polarisation of the Th17 response [35] that has an increasingly important role in the host response to *Candida* infection [36]. Netea *et al.* [37] have shown that neutrophil recruitment and phagocytosis of *Candida* are impaired in animals lacking TNF-alpha. Furthermore, TNF-alpha is an essential molecule for the successful control of infection and the development of a Th1-dependent response. IL-6 plays an important role in innate defense against *C. albicans* by recruiting neutrophils to the site of infection and IL-6-deficient mice are more susceptible to disseminated candidiasis than wild-type mice [38,39]. 

Despite the induction of innate and Th responses, infiltration of *C. albicans* is still prominent in the kidney and brain, which indicates that an ineffective host immune response is occurring. This could be due to the continuous expression of high levels of IL-10, an anti-inflammatory cytokine that participates in the immune dysfunction characteristic of sepsis [40,41] and induction of regulatory responses in kidney and brain which may restrict the immune response to *C. albicans*, and allow the infection to progress at these sites. IL-10 is a potent inhibitor in the immune defense against *C. albicans*. IL-10 inhibits phagocytic activity of human neutrophils [42] and blocks the release of pro-inflammatory cytokines, such as IL-1 and tumor necrosis factor alpha [43,44] in human monocytes. On the other hand, IL-10 is crucial for the generation of Treg cells [45] where Treg cells will stimulate the production of IL-4, IL-10, and TGF-β, which can inhibit inflammatory Th1 and Th17 responses [46]. In contrast, as *C. albicans* is steadily eliminated from the spleen without progression of infection, most of the PRR genes, cytokines and chemokines expression were suppressed at 72 h post-infection. The suppressive response seen in spleen by IL-10 accompanied with the shut down of innate and Th responses can avoid provocation of excessive immune responses which leads to tissue damage. Hence, we surmise that there is a balance in immune responses occurring in spleen and early immune response elicited in spleen can effectively prevent the overgrowth and colonization of *C. albicans* in this tissue.

The varied local host profiles during systemic *C. albicans* infection could be of importance for future work in designing targeted immunotherapy. Targeted immunotherapy involves the use of biological molecules or compounds to modulate immune responses in combination with drugs. Modulation of immune responses includes stimulation of host effectors immune responses that restore the impaired effector functions and inhibition of molecular pathways that are crucial for *Candida* growth and maintenance. Hence, attempts to identify mediators that are beneficial during systemic *C. albicans* infection are good choices of compounds for combination with current anti-*Candida* drugs to test for efficacy in treating systemic *C. albicans* infection. Likewise, the findings of local host immune responses among various organs in this study could serve as a basis for selection of potential beneficial mediators for immunotherapy study in various experimental models. 

This study has some limitations such as small sample sizes (animals per group) used for the gene expression study, the use of a single strain of *C. albicans* isolated from a healthy individual and the transcription profiling limited to a targeted inflammatory pathway that only includes the commonly expected pattern recognition receptors, chemokines and cytokines. We recognize the importance of broadening our search in future to other inflammatory and non-inflammatory biomarkers not included in this analysis and to identify the subset(s) of cells which is (are) responsible for the expression of immune response towards systemic *C. albicans* infection.

## 3. Experimental Section

### 3.1. Ethical Statement

All animal experiments were performed according to the guidelines in the Guide for the Care and Use of Laboratory Animals of the UPM Health Campus Animal Ethics Committee and approved by Animal Care and Use Committee (IACUC), Faculty of Medicine and Health Sciences, Universiti Putra Malaysia (UPM/FPSK/PADS/BR/UUH/00486). 

### 3.2. Mice

Six-week-old female BALB/c mice (weighing 20–25 g) were used for all animal experiments. The animals were randomized, assigned to groups of 6–8 mice and were given food and water *ad libitum*.

### 3.3. Fungal Inoculum and Animal Inoculation

The clinical isolate of *C. albicans* (HVS6360) used in this study originated from the vaginal swab of an immunocompetent patient who had vaginal candidiasis. *C. albicans* was grown on Sabouraud dextrose agar (SDA). For preparation of the inocula, *C. albicans* was quantified from SDA plates that had been incubated for 48 h at 35 °C and resuspended in phosphate buffer saline at the desired concentration. Female Balb/c mice were inoculated with *C. albicans* (5 × 10^5^ organisms/mouse) intravenously via lateral tail vein.

### 3.4. Quantitative Yeast Count

At 24, 72 and 168 h (days 1, 3, and 7) post infection (p.i) with *C. albicans*, three mice were euthanized, and target organs (kidney, brain, lung, spleen and liver) were excised for fungal burden determination and homogenized in 5 mL of sterile phosphate-buffered saline. Tissue homogenates from individual mice were serially diluted and inoculated on SDA plates and incubated for 48 h at 35 °C prior to quantification of *C. albicans*. The results were expressed as log_10_ CFU/gram of tissues.

### 3.5. Determination of Haemolysin Activity

Haemolysin activity of clinical *C. albicans* isolate (HVS6360) was evaluated with a blood plate assay as described previously [47]. A loopful of an overnight yeast culture (approximately 10^8^ cells/mL determined through cell counting using a haemocytometer) was aseptically deposited onto the medium and the plate was then incubated at 37 °C in 5% CO_2_ for 48 h. *Candida albicans* ATCC140154 strain was used as positive control in this study.

After incubation, the diameter of the colony and the diameter of the translucent zone of haemolysis were measured. The ratio of *x*/*y*, which represent haemolysis index (HI value) was measured, where *x* is the total diameter of the translucent zone plus the colonies, and y is the diameter of the colonies. 

### 3.6. Red Blood Cells and Haemoglobin Counts

For blood profile, 0.2 mL of whole blood was drawn from mice at 0, 72 and 168 h (days 0, 3 and 7) post infection via cardiac puncture. Blood samples were subjected to full blood analysis by using the automated hematology analyzer (Sysmex KX-21, Sysmex Corporation, Kobe, Japan) and the changes in the red blood cells and haemoglobin counts were monitored and recorded. Changes in red blood cells and haemoglobin counts between 0 h (pre-infected stage) and 72 h and 168 h post infection were analyzed by Mann-Whitney test using GraphPad Prism 5 (GraphPad Software, Inc., San Diego, CA, USA); *p*-values <0.05 were considered statistically significant.

### 3.7. Erythropoietin Expression

Real time RT-PCR assay was performed to specifically quantify murine erythropoietin. Briefly, kidneys were excised and blood was drawn from *C. albicans* infected mice at 72 h post-infection. Total RNA from kidney and blood was extracted using high pure RNA tissue kit (Roche, Mannheim, Germany) and AquaPure RNA Isolation kit (Biorad, Hercules, CA, USA) according to manufacturer’s directions and stored at −80 °C prior to analysis. RNA integrity was checked by running normal agarose gel electrophoresis and RNA concentration was quantified by NanoDrop^®^ ND-1000A spectrophotometer (NanoDrop Technologies Inc., Wilmington, DE, USA). Isolated RNA (500 ng) was reversed transcribed to cDNA with iScript reverse transcription supermix (Biorad). Samples were then subjected to two step amplification of 40 cycles using SsoFast Evagreen Supermix (Biorad) with cycling condition: initial denaturation 95 °C for 3 min, followed by denaturation; 95 °C for 15 s, annealing; 61 °C for 3 s in the CFX96 Miniopticon detection system (Biorad, Foster City, CA, USA) as specified by the manufacturer. PCR amplification of beta-actin and GAPDH were performed for each sample and to allow normalization between samples. Water controls (non-template controls) were included to ensure specificity. The murine erythropoietin expression at 72 h post infection was compared with the uninfected groups and was analyzed by Mann-Whitney test. A *p <* 0.05 value was considered as statistically significant.

### 3.8. Pathology

The inflammatory response and presence of yeast infiltration in target organs of mice infected systemically with *C. albicans* were assessed by light microscopy. At 72 h (day 3) post-infection, groups of three surviving mice were euthanized, and tissues were excised and fixed in 10% buffered formalin. Fixed tissues were subjected to tissue processing, embedded with paraffin, sectioned and stained with haematoxylin-eosin (H&E) and periodic acid Schiff (PAS) stain. 

### 3.9. Local Host Immune Response

Customized PCR array (CAPM11464, SA Biosciences, Qiagen, Frederick, MD, USA) (Table 1) was used to analyze the expression of 32 key genes, including 25 genes involved in inflammatory immune response, four housekeeping genes and controls to check for genomic DNA contamination, RNA quality and general PCR performance (Table 1). Briefly, kidneys, spleen and brain were excised from groups of three mice from both the uninfected group and the groups infected with *C. albicans* at 24 and 72 h post infection and organs were stored in RNAlater solution (Ambion, Inc., Austin, TX, USA) prior to extraction. Total RNA was extracted using high pure RNA tissue kit (Roche) according to manufacturer’s directions and stored at −80 °C prior to analysis. RNA integrity was checked by running normal agarose gel electrophoresis and RNA concentration was quantified by NanoDrop^®^ ND-1000A spectrophotometer (NanoDrop Technologies Inc.). Isolated RNA (500 ng) samples were reversed transcribed into cDNA with the RT^2^ PCR Array First Strand Kit (SA Biosciences). cDNA were then mixed with ready-to-use RT^2^ Real-Time™ SYBR Green PCR Master Mix (SA Biosciences). Subsequently, 25 μL aliquots of this mixture was added to each well of the same PCR Array plate containing the pre-dispensed gene-specific primer sets, and PCR Array was performed by using Mastercycler Eppendorf Realplex4 PCR machine (Eppendorf, Hamburg, Germany). The cycling conditions for PCR Array was as follows: 10 min pre-denaturation at 95 °C, followed by 40 cycles of denaturation for 15 s at 95 °C and annealing for 1 min at 60 °C. Dissociation (melting curve) analysis was performed after each run to ensure PCR specificity. Threshold cycle (*C*_t_) values for all the genes on each PCR Array were calculated and fold changes in gene expression for pair-wise comparison were calculated by using the ΔΔ*C*_t_ method. 

### 3.10. Data and Statistical Analysis

The PCR data were uploaded to SA Biosciences Web Analysis [48] for analysis. Threshold cycle (*C*_t_) cut-off was set at 35 cycles. Fold-change values greater than zero indicate an up-regulation, and fold-change values less than zero indicate a down-regulation, and the fold-regulation is the negative inverse of the fold-change. Comparison of the gene expression data between infected organs and control (uninfected organs) were analyzed with Mann-Whitney test using GraphPad Prism 5; *p*-values <0.05 were considered statistically significant.

## 4. Conclusions

The pathogenesis of systemic *C. albicans* infection could involve a multistep process which subsequently leads to death of the infected mice due to progressive sepsis in targeted organs. Reduction of red blood cells and haemoglobin levels throughout the infection period could be associated with the haemolysin production by *C. albicans*. The differential profiles of PRRs, chemokines, and cytokines seen in the kidney, spleen and brain suggest that local host immune responses might vary among target organs during systemic *C. albicans* infection. This has crucial implications for future exploratory research of targeted therapies against this opportunistic pathogen using immunomodulatory approaches. 
